# Bulk and Single-Cell Transcriptomes Reveal Exhausted Signature in Prognosis of Hepatocellular Carcinoma

**DOI:** 10.3390/genes16091034

**Published:** 2025-08-30

**Authors:** Ruixin Chun, Haisen Ni, Ziyi Zhao, Chunlong Zhang

**Affiliations:** College of Computer and Control Engineering, Northeast Forestry University, Harbin 150040, China; 2022224532@nefu.edu.cn (R.C.); 15945063693@163.com (H.N.); wobushizzy@gmail.com (Z.Z.)

**Keywords:** hepatocellular carcinoma, T cell exhaustion, tumor microenvironment, scRNA-seq

## Abstract

Background/Objectives: Hepatocellular carcinoma (HCC) is a highly heterogeneous malignancy with poor prognosis. T cell exhaustion (TEX) is a key factor in tumor immune evasion and therapeutic resistance. In this study, we integrated single-cell RNA sequencing (scRNA-seq) and bulk RNA sequencing (RNA-seq) data to characterize TEX-related transcriptional features in HCC. Methods: We first computed TEX scores for each sample using a curated 65-gene signature and classified them into high-TEX and low-TEX groups by the median score. Differentially expressed genes were identified separately in scRNA-seq and bulk RNA-seq data, then intersected to retain shared candidates. A 26-gene prognostic signature was derived from these candidates via univariate Cox and LASSO regression analysis. Results: The high-TEX group exhibited increased expression of immune checkpoint molecules and antigen presentation molecules, suggesting a tumor microenvironment that is more immunosuppressive but potentially more responsive to immunotherapy. Functional enrichment analysis and protein–protein interaction (PPI) network construction further validated the roles of these genes in immune regulation and tumor progression. Conclusions: This study provides a comprehensive characterization of the TEX landscape in HCC and identifies a robust gene signature associated with prognosis and immune infiltration. These findings highlight the potential of targeting TEX-related genes for personalized immunotherapeutic strategies in HCC.

## 1. Introduction

Primary liver cancer, including hepatocellular carcinoma (HCC) and intrahepatic cholangiocarcinoma (ICC), has been a major global health issue due to its high mortality and rising incidence. In 2020, there were an estimated 905,700 new cases and 830,200 deaths, making it the third leading cause of cancer deaths worldwide. By 2040, new cases are predicted to increase by 55% [1]. Liver cancer epidemiology was affected by factors such as hepatitis B and C, alcohol, and metabolic disorders. The Global Burden of Disease Study showed that although global age-standardized incidence and death rates for liver cancer were declining, the total number of cases and deaths was rising due to population growth and aging [2]. The overall 5 year survival rate for individuals with HCC was alarmingly low, approximately 20% [3,4,5,6]. The tumor microenvironment (TME), particularly its highly complex and heterogeneous characteristics, played a key role in cancer progression and therapeutic resistance, thereby constituting a crucial target for enhancing patient prognosis [7,8,9]. The heterogeneity inherent in tumor biology and patient responses to treatment presented a substantial challenge within the field of oncology. This variability was especially pronounced in the context of adjuvant therapies, wherein identical treatments could result in markedly disparate outcomes among patients [10].

T cell exhaustion (TEX), a state of T cell dysfunction that was commonly observed in both chronic infections and cancer, has garnered significant attention in recent years [11]. It was characterized by the sustained expression of inhibitory receptors on the T cell surface and a progressive loss of effector functions [12,13]. Exhausted T cells had been identified as critical components within the immunosuppressive landscape of HCC. They played an important role in facilitating tumor immune escape and contributing to resistance against various therapeutic interventions [14,15]. However, our understanding of the heterogeneity and functional plasticity of TEX cells within the HCC microenvironment remained incomplete.

The advent of single-cell RNA sequencing (scRNA-seq) technology had revolutionized our ability to investigate the cellular composition and functional states of tumors [16]. The capacity to dissect transcriptional profiles and phenotypic diversity of individual cells within the tumor milieu represents a significant advancement in cancer research, offering insights into the complex ecosystem of tumors. Compared to traditional bulk transcriptomic analysis, scRNA-seq provided a fine-grained, high-resolution analysis of TEX subpopulations [17]. It had the potential to uncover novel functional states and intercellular interactions that may have been overlooked by previous approaches. Over the preceding years, several studies have explored immune-related prognostic models for HCC [18]. For example, Wang et al. developed a 10-gene immune-related prognostic signature [19], and Liu et al. subsequently proposed a 6-gene immune-related model [20], both of which were constructed exclusively from bulk transcriptomic data. However, most of these models either did not specifically address the transcriptional complexity of exhausted T cells or lacked robustness across data modalities.

**Figure 1:**
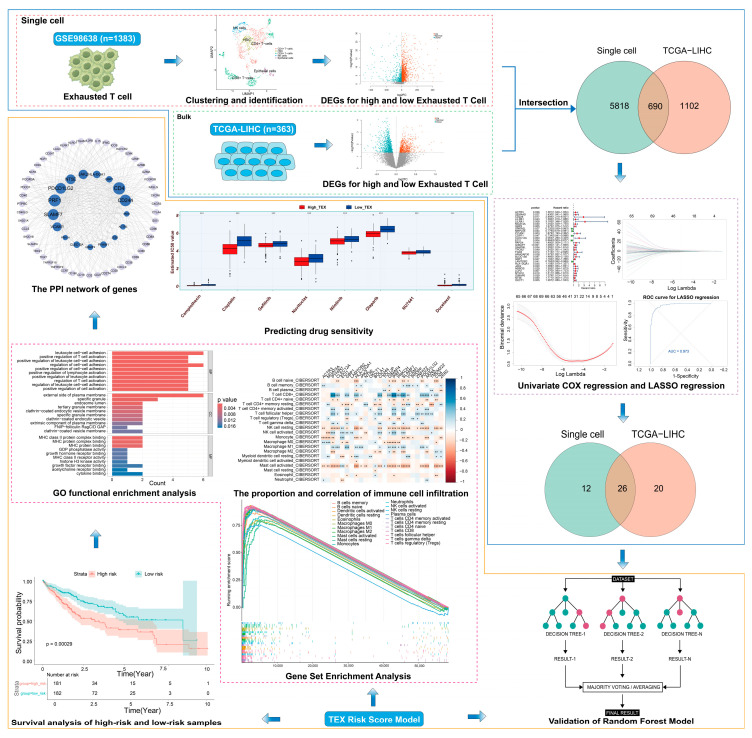
The workflow of this study.

In this study, we integrated scRNA-seq and bulk RNA-seq data to characterize exhausted T cells (TEX) in HCC (Figure 1). Using the least absolute shrinkage and selection operator (LASSO) regression analysis for feature selection, we identified TEX-associated signatures to construct a prognostic scoring system and explored mechanisms underlying high-TEX and low-TEX groups, including immune infiltration, oncogenic pathways, and protein–protein interaction (PPI) networks.

## 2. Materials and Methods

### 2.1. Data Acquisition

The HCC scRNA-seq data (GSE98638) were obtained from the Gene Expression Omnibus (GEO) database (https://www.ncbi.nlm.nih.gov/geo (accessed on 28 March 2024)). From this dataset, we specifically extracted 1383 exhausted T cells for downstream analysis based on the cell-type annotations provided in the original publication, comprising 23,389 genes. The expression profile was provided in TPM (Transcripts Per Million) format. Further single-cell analysis was performed using the Seurat (v5.3) package in R. The Bulk RNA-seq data of liver hepatocellular carcinoma were obtained from The Cancer Genome Atlas (TCGA) database (https://portal.gdc.cancer.gov) (TCGA-LIHC), including 363 tumor samples and 23,389 genes. The expression profile was provided in FPKM (Fragments Per Kilobase of transcript per Million mapped reads) format. Additional external validation datasets GSE76427 and GSE14520 were also downloaded from GEO, including 115 and 221 tumor samples, respectively. The expression profile was provided in FPKM format.

### 2.2. Classification Based on Exhausted T Cell Signatures

A total of 65 TEX-related marker genes were downloaded from the CellMarker database [21]. For single-cell data, the AUCell (v1.30) package in R was used to calculate TEX scores at the cellular level and single sample gene set enrichment analysis (ssGSEA) was applied to compute TEX scores in bulk RNA-seq samples [22]. Cells and patients were stratified into high-TEX and low-TEX groups based on the median TEX score within each dataset.

### 2.3. Identification of Differentially Expressed Genes

To identify genes associated with TEX, differential expression analysis was performed between high-TEX and low-TEX groups. The Wilcoxon rank-sum test was applied to single-cell data (GSE98638), and the limma (v3.64) package in R was used for bulk data (TCGA-LIHC). The differentially expressed genes (DEGs) with a *p* value ≤ 0.05 were retained. The intersection of DEGs from both datasets was selected as a candidate TEX gene signature for subsequent analysis.

### 2.4. Construction of the TEX Risk Score Model

Univariate Cox regression analysis was used to screen prognosis-related genes (*p* value ≤ 0.05). Subsequently, the LASSO regression analysis was independently conducted on both single-cell and bulk RNA-seq datasets to identify key genes distinguishing high-TEX and low-TEX groups. The overlapping genes from both datasets were defined as the TEX gene signature.

### 2.5. Establishment and Validation of the TEX Diagnostic Model

A diagnostic model based on the random forest algorithm was constructed using the randomForest (v4.7) package in R. We set the number of trees to 500, which ensured both stability and computational efficiency, and enabled feature importance estimation. Model hyperparameters were optimized through grid search combined with ten-fold cross-validation on the training datasets. GSE98638 and TCGA-LIHC were used as training datasets, and GSE14520 and GSE76427 were served as independent validation datasets. The model performance was evaluated using the area under the curve (AUC) calculated by the pROC (v1.18) package in R.

### 2.6. Functional Enrichment Analysis

Gene Ontology (GO) and Kyoto Encyclopedia of Genes and Genomes (KEGG) enrichment analysis were performed using the clusterProfiler (v4.16) package in R. Terms with *p* value ≤ 0.05 were considered significantly enriched. GSEA was also carried out using clusterProfiler based on the CIBERSORT reference gene set [23]. The gene set included 22 human immune cell types. Genes were ranked according to fold change (mean of high-TEX group/mean of low-TEX group) and subjected to 1000 permutation tests to obtain nominal *p* values. Gene sets with *p* value ≤ 0.05 were considered statistically enriched.

### 2.7. Construction of PPI Network

The key genes were submitted to the STRING database (https://string-db.org), and PPI pairs involving these key genes were identified using the following parameters: (1) full STRING network, (2) edge meaning set to confidence, (3) active interaction sources including experiments, text mining, databases, co-expression, neighborhood, gene fusion, and co-occurrence, (4) minimum required interaction score = 0.4, (5) maximum number of interactors set to “query proteins only.” The PPI network was constructed and visualized in Cytoscape (v3.7.2), and hub genes were identified using the cytoHubba plugin.

## 3. Results

### 3.1. Grouping of Cells and Samples Based on TEX Genes

In this study, we focused on the analysis of transcriptomic data to investigate the characteristics of exhausted T cells and their distribution across different immune cell populations. We selected a 65 TEX-related gene signature from the CellMarker database for further analysis.

**Figure 2:**
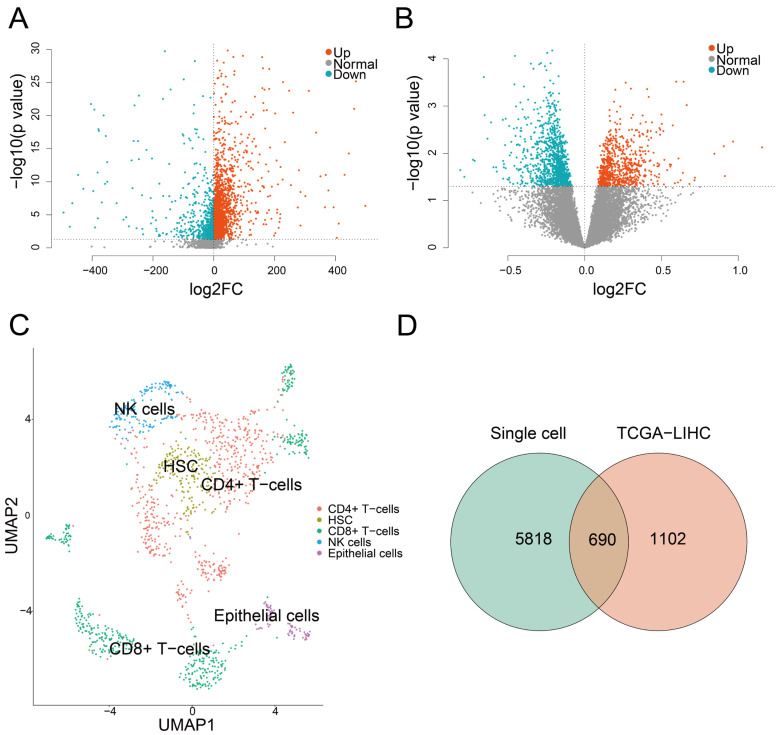
The result of differential expression analysis. (**A**) A volcano plot of differential expression analysis for GSE98638. (**B**) A volcano plot of differential expression analysis for TCGA. The red and blue color represent the significantly up-regulated and down-regulated genes. The *x*-axis represents the log2 fold change (log2FC), and the *y*-axis represents the −log10(*p* value) from the differential expression analysis. (**C**) The UMAP result of GSE98638 showing the annotation and color codes for 5 cell types in HCC. (**D**) A venn diagram of single-cell and bulk DEGs.

To characterize exhaustion at the single-cell level, we extracted immune cells from tumor-derived scRNA-seq data (GSE98638). After preprocessing, 1383 cells and 23,389 genes were retained. Clustering analysis was performed using the Seurat package, followed by dimensionality reduction via uniform manifold approximation and projection (UMAP). Reference datasets for cell annotation were obtained using the celldex (v1.18) package in R, and annotation was conducted with SingleR, identifying five major cell types: CD4^+^ T cells, CD8^+^ T cells, hematopoietic stem cells (HSCs), natural killer cells (NKs), and epithelial cells (Figure 2C).

In order to quantify the exhaustion status of individual cells, we calculated the TEX score for each cell based on the selected gene set using the AUCell package. Cells were stratified into high-TEX and low-TEX groups using the median TEX score. Differential expression analysis was conducted using the Wilcoxon rank-sum test. Genes with *p* value ≤ 0.05 and log2FC > 0 were classified as up-regulated, and those with *p* value ≤ 0.05 and log2FC < 0 were considered down-regulated, where log2FC represents the log2-transformed fold change calculated as the mean expression in the high-TEX group divided by the mean expression in the low-TEX group. A total of 6508 DEGs were identified, including 4316 up-regulated and 2192 down-regulated genes (Figure 2A). Next, we further calculated TEX score based on bulk RNA-seq data from 363 liver cancer (TCGA-LIHC) patients, encompassing expression profiles of 23,389 genes. The ssGSEA algorithm from the GSVA (v2.2) package was applied to calculate TEX score per sample. Samples were grouped into high-TEX and low-TEX groups based on the median score. Differential expression analysis between the two groups was assessed using the limma package in R. Genes with *p* value ≤ 0.05 and log2FC > 0 were defined as up-regulated, and those with *p* value ≤ 0.05 and log2FC < 0 were considered down-regulated. A total of 1792 DEGs were identified, including 662 up-regulated and 1130 down-regulated genes (Figure 2B). Then we determined the intersection of DEGs derived from both single-cell and bulk RNA-seq data, resulting in 690 genes (Figure 2D).

### 3.2. Prognosis of T Cell Exhaustion Gene Signature

**Figure 3:**
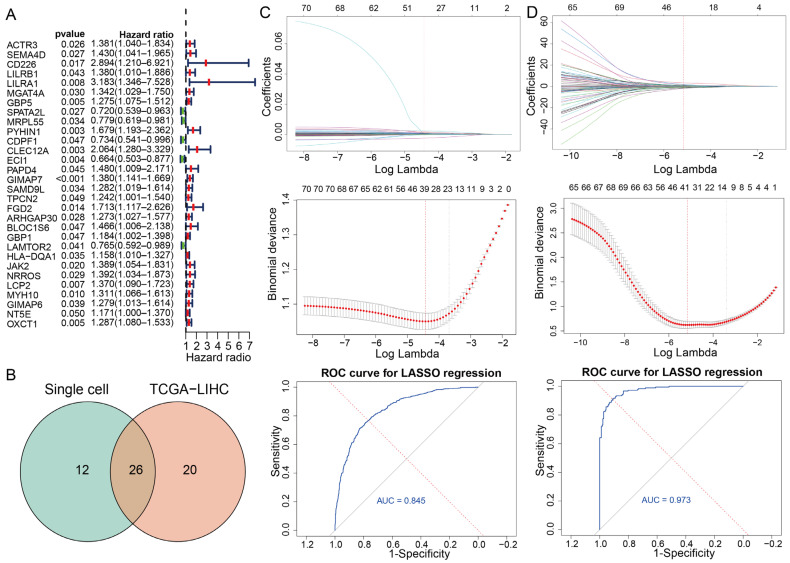
Identification of TEX-related prognostic genes. (**A**) The significant result of univariate Cox regression analysis on 690 genes. The red and green box represent positive and negative association with risk event, respectively. (**B**) The intersection of robust marker genes between Single cell and TCGA-LIHC. (**C**) Identification of biomarkers in single-cell data. (**D**) Identification of biomarkers in bulk data. The first panel represents the LASSO coefficient path for risk factors. The second panel represents the cross-validation curve. The third panel represents the AUC of LASSO regression analysis.

To further assess their prognostic relevance, we employed univariate Cox regression analysis on these 690 genes, which led to the identification of 71 genes significantly associated with overall survival (*p* value ≤ 0.05) (Figure 3A and Supplementary Figure S1).

To enhance the identification of TEX-related genes with prognostic significance, we employed the LASSO regression analysis on both scRNA-seq and bulk RNA-seq datasets. The LASSO regression analysis of the single-cell dataset identified 38 candidate genes (Figure 3C), and the analysis of the TCGA-LIHC cohort identified 46 candidate genes (Figure 3D). The intersection of these two gene sets yielded 26 robust markers, which were subsequently designated as TEX-related prognostic genes (Figure 3B).

**Figure 4:**
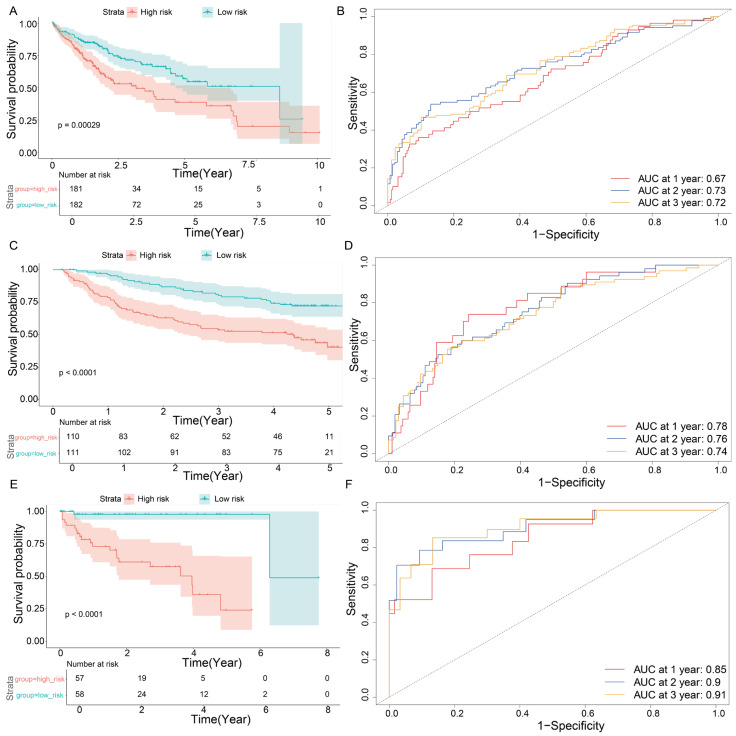
Survival analysis. (**A**) The survival result of TCGA. (**B**) The AUC of TCGA. (**C**) The survival result of GSE76427. (**D**) The AUC of GSE76427. (**E**) The survival result of GSE14520. (**F**) The AUC of GSE14520. The top figure of the survival result represents the survival curve, and the bottom figure of the survival result represents the number of survivors at different time points.

To further estimate the importance of 26 genes in HCC prognosis, we developed a multivariate Cox regression analysis to compute a risk score for each patient. Subsequently, patients were categorized into high-risk and low-risk groups according to the median risk score. Survival analysis revealed that the high-risk group exhibited significantly poorer overall survival compared to the low-risk group (*p* = 0.00029) (Figure 4A). To further assess the predictive efficacy of the gene signature, we performed a time-dependent receiver operating characteristic (ROC) analysis. The gene set demonstrated robust discriminatory capability for 1, 2, and 3 year survival, with AUC values of 0.67, 0.73, and 0.72, respectively (Figure 4B). These findings suggested potential clinical utility of our pipeline in prognostic prediction.

To assess the robustness and generalizability of the identified signature, we conducted external validation utilizing two independent GEO datasets, GSE76427 and GSE14520. Consistent with our expectations, survival analysis based on the 26-gene signature effectively stratified patients into high-risk and low-risk groups, demonstrating significant differences in survival outcomes (GSE76427, *p* value ≤ 0.0001; GSE14520, *p* value ≤ 0.0001) (Figure 4C,E). Furthermore, ROC curve analysis corroborated the strong predictive performance of the signature for 1, 2, and 3 year survival across both datasets (GSE76427, AUC = 0.85, 0.90, 0.91; GSE14520, AUC = 0.78, 0.76, 0.74) (Figure 4D,F). These results demonstrated that the identified gene signature not only reflected TEX status but also served as a reliable predictor of patient prognosis in HCC across multiple datasets.

### 3.3. The Predictive Modeling of the T Cell Exhaustion Gene Signature

**Figure 5:**
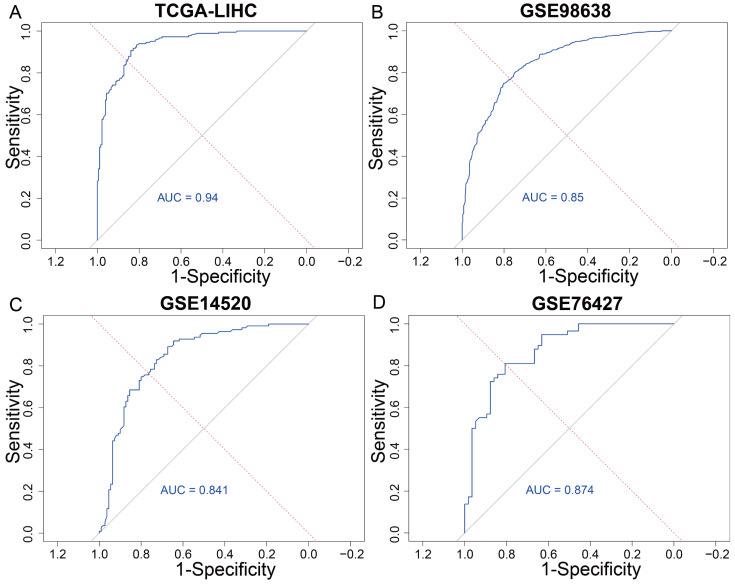
The predictive result of TEX gene signature. (**A**) The AUC of TCGA. (**B**) The AUC of GSE98638. (**C**) The AUC of GSE14520. (**D**) The AUC of GSE76427.

In order to further validate the predictive power of the identified TEX gene signature in assessing patient risk, we employed a machine learning-based classification approach using the random forest algorithm combined with ten-fold cross-validation. Classification models were constructed using both the TCGA-LIHC dataset and the GSE98638 single-cell dataset, with the 26 TEX genes as input features and exhaustion risk (high vs. low) as the classification label. The random forest model trained on the TCGA dataset achieved a high average AUC of 0.94 (Figure 5A), indicating good classification performance. Similarly, the model on the GSE98638 dataset achieved an average AUC of 0.85 (Figure 5B), further supporting the predictive capacity of the TEX gene signature in an independent single-cell dataset.

To assess the stability of the model, external validation was performed using two additional independent datasets. On GSE14520 and GSE76427, the model reached an average AUC of 0.819 and 0.876 (Figure 5C,D). These results confirm that the TEX gene signature serves as a robust and reliable biomarker set for predicting TEX risk across diverse patient populations and data modalities. These findings demonstrate the strong potential of the machine learning-based model, not only in accurately classifying exhaustion risk but also in offering value for prognostic evaluation and personalized treatment stratification in HCC.

### 3.4. Functional Enrichment Analysis of the T Cell Exhaustion Gene Signature

**Figure 6:**
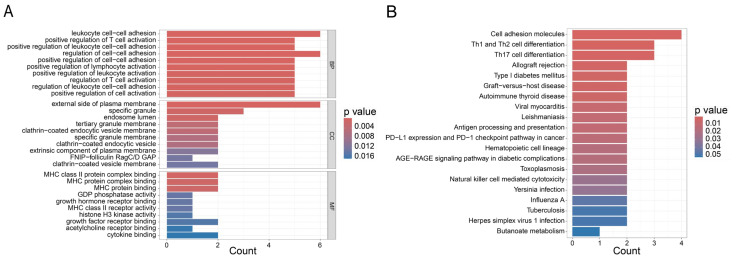
Functional enrichment result of TEX gene signature. (**A**) GO analysis. The top barplot represents the result of BP. The middle barplot represents the result of CC. The bottom barplot represents the result of MF. (**B**) The result of KEGG. The count of each barplot represents the number of genes enriched in function, and the color represents the *p* value.

In order to explore the potential biological roles of the 26-gene exhaustion signature, we performed functional enrichment analysis using the Enrichr platform [24]. The GO enrichment results revealed a total of 265 significantly enriched biological process (BP) terms, 28 cellular component (CC) terms, and 37 molecular function (MF) terms (Figure 6A). We selected the top 10 representative terms from each category for detailed presentation. Among the BP terms, the enriched terms included leukocyte adhesion, positive regulation of T cell activation, positive regulation of leukocyte adhesion, regulation of adhesion, positive regulation of adhesion, positive regulation of lymphocyte activation, positive regulation of leukocyte activation, regulation of T cell activation, and regulation of leukocyte adhesion. These processes are central to immune cell homing, positioning, and interactions with target cells—particularly involving adhesion molecules critical for leukocyte trafficking and transmigration during inflammatory responses. Moreover, the positive regulation of T cell and lymphocyte activation underscores the role of the signature in initiating and amplifying adaptive immune responses. Among the CC terms, the enriched terms included external side of plasma membrane, specific granule, endosome lumen, tertiary granule membrane, clathrin-coated endocytic vesicle membrane, and extrinsic component of plasma membrane. These components were important to immunological signaling, molecular transport, and the release of effector molecules. For instance, specific granules were known to store and secrete a variety of bioactive compounds that regulate immune and inflammatory responses [25]. Among the MF terms, the enriched terms included major histocompatibility complex (MHC) class II protein complex binding, GDP phosphatase activity, growth hormone receptor binding, histone H3 kinase activity, cytokine binding, and acetylcholine receptor binding. These functions highlighted the involvement of the gene set in antigen presentation, signal transduction, transcriptional regulation, and immune modulation. Notably, MHC binding and cytokine interactions were fundamental to T cell-mediated immunity and inflammatory signaling. From an oncological perspective, regulation of cell adhesion processes was tightly linked to tumor progression and metastasis. Aberrant expression of adhesion molecules could facilitate tumor cell detachment, invasion, and dissemination. Cellular components such as the plasma membrane and endocytic vesicles were also implicated in altered receptor signaling and nutrient acquisition in cancer cells. On the molecular function level, enrichment in growth factor receptor binding and histone kinase activity suggested involvement in proliferative signaling and epigenetic regulation—both of which are key drivers of tumor growth and therapy resistance.

The Enrichr identified a total of 16 significantly enriched KEGG pathways, many of which were closely linked to cancer, immune responses and TEX. Notably enriched pathways such as “cell adhesion molecules, antigen processing and presentation, PD-L1 expression, and PD-1 checkpoint pathway in cancer” highlighted key aspects of immune regulation and tumor interaction (Figure 6B). Cell adhesion molecules play a key role in immune cell migration and tumor cell interaction. They facilitate the homing of immune cells to tumor sites and mediate the binding and killing of tumor cells by immune cells. In cancer, abnormal expression of these molecules may promote tumor cell detachment and metastasis. Antigen processing and presentation were essential for activating T cells. Tumor cells often downregulated antigen presentation to evade immune detection. The enrichment of this pathway underscored the interaction between tumor cells and the immune system in the context of antigen recognition. The PD-L1/PD-1 pathway was a critical immune checkpoint. Its activation could lead to TEX, enabling tumor cells to evade immune surveillance. The enrichment of this pathway suggested a potential mechanism for immunotherapy targeting this checkpoint to restore T cell function and combat cancer.

In summary, the functional enrichment analysis revealed significant association between the exhaustion signature and key biological processes related to cancer, immune responses, and TEX. The results highlight the interplay between immune cell interactions, adhesion, activation, and antigen presentation, as well as the critical pathways involved in cancer progression and immune regulation. These findings provide a foundation for further exploration of potential immunotherapy targets and the complex mechanisms underlying cancer-immune interactions.

### 3.5. Analysis of Immune Infiltration for the T Cell Exhaustion Gene Signature

**Figure 7:**
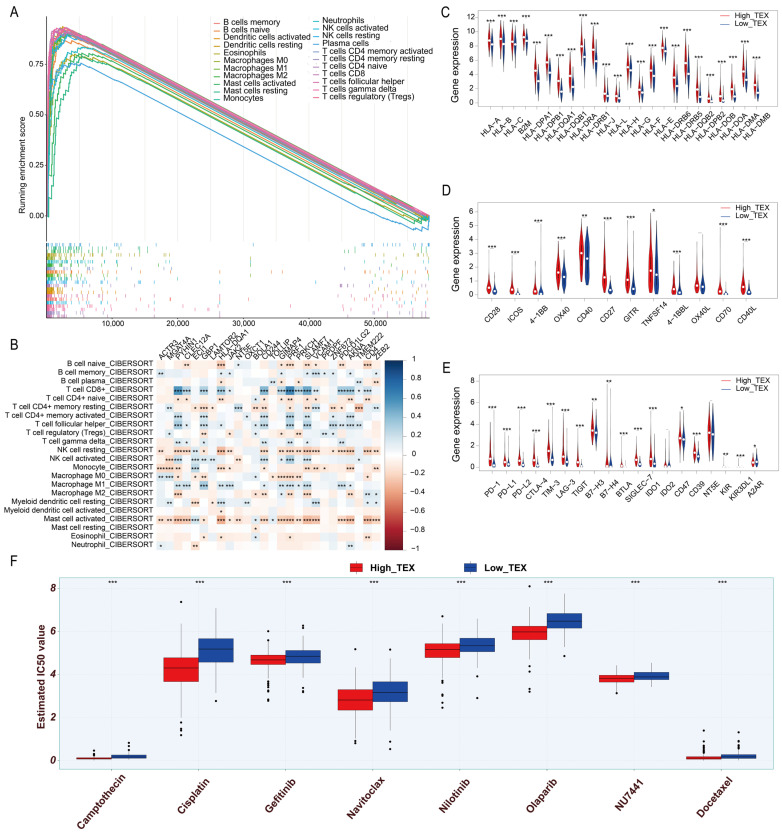
The immune result for TEX gene signature. (**A**) The GSEA result of immune infiltration. (**B**) Correlation coefficient between signature and immune infiltration. The color represents the Pearson correlation coefficient. (**C**) Antigen presentation expression between high-TEX and low-TEX group. (**D**) Costimulatory signal expression between the high-TEX and the low-TEX group. (**E**) Immune checkpoint molecule expression between the high-TEX and the low-TEX group. (**F**) Drug sensitivity between the high-TEX and the low-TEX group. “***”, “**” and “*” denote *p*-values of ≤0.001, 0.01, and 0.05 in (**C**–**F**).

In order to further investigate the association between cancer prognosis and immune activity, we conducted GSEA analysis to explore the relationship between TEX-related risk groups and immune infiltration. Genes were ranked based on fold changes between the high-risk and low-risk groups, followed by GSEA analysis. The results revealed significant enrichment of immune infiltration gene signatures associated with 22 immune cell types (Figure 7A).

To gain deeper insights into the immunological relevance of the 26-gene exhaustion signature, we further analyzed the Pearson correlation between individual gene and immune cell infiltration levels. Among them, eight genes—*PYHIN1*, *GBP1*, *HLA-DQA1*, *CD244*, *GIMAP4*, *PRF1*, *SLAMF7*, and *AKNA*—were found to be significantly correlated with more than ten distinct immune cell types (Figure 7B). *PYHIN1*, a member of the interferon-inducible *HIN-200* protein family, was found to play a critical role in innate immunity by detecting cytoplasmic double-stranded DNA and activating antiviral responses. Notably, *PYHIN1* was also shown to modulate pro-inflammatory cytokine expression in airway epithelial cells, such as *IL-6* and *TNFα*, in response to *IL-1* or *TNFα* stimulation [26,27]. *GBP1* had been implicated in immune escape and immunotherapy responsiveness. Its expression had been identified as a predictive biomarker in *IFN*-related pathways (*IFN-α/β/γ*, *IL-15*), particularly in the context of CD8^+^ T cell activation [28]. *GBP1* was also found to facilitate immune evasion by promoting extracellular secretion of indoleamine 2,3-dioxygenase, thereby enhancing tumor malignancy [29]. *HLA-DQA1* was linked to immunogenicity risk in immune-mediated inflammatory disorders. Carriers of the *HLA-DQA105* allele exhibited increased immunogenicity when treated with TNFα antagonists [30], indicating the potential relevance of HLA-DQA1 to immune modulation in cancer therapy. *CD244* encodes an immunoregulatory receptor expressed in NK cells, T cells, dendritic cells, and myeloid-derived suppressor cells. *CD244* expression was elevated in exhausted immune cells within the TME, contributing to diminished effector function [31]. *GIMAP4* was identified as a critical regulator of T cell differentiation and cytokine secretion, particularly that of *IFN-γ*, during early CD4^+^ T cell development. Its loss downregulated VMA21, an endoplasmic reticulum-localized chaperone protein, suggesting its involvement in intracellular transport and immune modulation [32]. *PRF1* expression was suppressed in HCC via PRMT5-mediated methylation of *TCF12*, leading to upregulation of *FGL1* and impaired CD8^+^ T cell responses. PRMT5 inhibition enhanced PD-L1 blockade efficacy, suggesting its therapeutic potential in combination immunotherapy [33]. *SLAMF7* activation in T cells induces features of exhaustion. In clear cell renal cell carcinoma, elevated *SLAMF7* expression was associated with poor prognosis and a macrophage-dominant immune phenotype, indicating its potential as a therapeutic target to restore antitumor immunity [34]. *AKNA* has been reported to regulate inflammation, immune response, and transition (EMT) in epithelial ovarian cancer, highlighting its multifaceted role in tumor progression [35].

We further examined differences in drug sensitivity, antigen presentation, costimulatory signals, and immune checkpoint molecule expression between high-TEX and low-TEX groups using the Wilcoxon rank-sum test, analyzing their impact on prognosis. In the high-TEX group, several human leukocyte antigen (HLA) genes (e.g., *HLA-A*, *HLA-B*, *HLA-C*) exhibited significantly higher expression (*p* value ≤ 0.001), with median levels between 8 and 11, compared to 6 to 9 in the low-TEX group (Figure 7C). This elevated expression of antigen presentation molecules enhances tumor cell recognition by T cells, activating the immune system to target and destroy tumor cells. Costimulatory molecules are integral to the activation of T cells. Accordingly, in the high-TEX group, there was a notably elevated expression of prototypical costimulatory molecules, including *CD28*, *ICOS*, *4-1BB*, *OX40*, *CD40*, *CD27*, *GITR*, *TNFSF14*, *4-1BBL*, *OX40L*, *CD70*, and *CD40L* (Figure 7D). Elevated expression of these molecules provides the requisite signal for T cell activation and proliferation. Enhanced levels of *CD28*, *ICOS*, *4-1BB*, *OX40*, *CD40*, *CD27*, *GITR*, *TNFSF14*, *4-1BBL*, *OX40L*, *CD70* and *CD40L*, and that of other costimulatory molecules were strongly associated with more robust antitumor T cell responses [36].

Immune checkpoint molecules were typically considered to represent “immune inhibitory” markers. In the high-TEX group, expression levels of most immune checkpoint genes (including *PD-1/PD-L1/PD-L2*, *CTLA-4*, *TIM-3*, *LAG-3*, *TIGIT*, *B7-H3*, *B7-H4*, *BTLA*, *SIGLEC-7*, *IDO1*, *IDO2*, *CD47*, *CD39*, *NT5E*, *KIR*, *KIR3DL1*, and *A2AR*) were significantly elevated compared to the low-TEX group, with the majority of genes exhibiting *p* values less than 0.01 (Figure 7E). This observation suggests that the upregulation of immune checkpoint molecules was prevalent in TME characterized by highly active immune cells. Such upregulation may indicate heightened immune cell activity within the tumor microenvironment and imply that these patients potentially exhibit a more favorable response to immune checkpoint inhibitors, such as anti-PD-1/PD-L1 and anti-CTLA-4 therapies [37].

We systematically predicted the response of multiple chemotherapeutic agents routinely used in the treatment of HCC and compared the IC50 distributions across molecular subtypes. For several established HCC chemotherapeutics, the high-TEX subtype exhibited markedly lower IC50 values than the low-TEX subtype, indicating that high_TEX tumors are significantly more sensitive to these agents [38]. Conversely, low_TEX tumors displayed elevated IC50 values, suggesting an intrinsic resistance to standard chemotherapy and highlighting their potential for poorer prognosis and diminished response to conventional therapeutic regimens.

These findings suggest that TEX-related genes are linked to immune regulation and infiltrating immune cells, serving as potential prognostic biomarkers and immunotherapy targets in HCC and other cancers. Patients in the high-TEX group showed high expression of antigen presentation and costimulatory molecules, enhancing T cell activation and likely responding better to immunotherapy, leading to improved outcomes. In contrast, the low-TEX group had lower immune-related gene expression, resulting in poor immune infiltration and T cell activation, making them less responsive to immunotherapy and associated with a poorer prognosis.

### 3.6. The Construction of PPI Network

**Figure 8:**
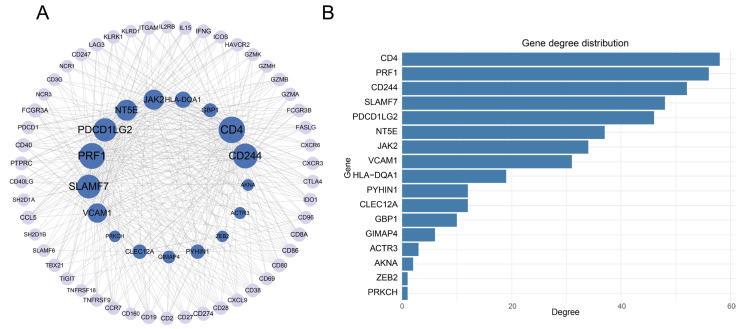
The PPI analysis of gene signatures. (**A**) The PPI network of gene signatures. The dark node represents the gene signatures. The light node represents the genes interacting with gene signatures. (**B**) The degree of gene signatures.

In order to further validate the functional roles of the exhaustion-associated gene signature, we constructed a PPI network using the 26 identified genes. The resulting subnetwork consisted of 67 nodes and 405 edges, with 17 of the 26 gene signature present (Figure 8A). Notably, five of these genes—*CD4*, *PRF1*, *CD244*, *SLAMF7*, and *PDCD1LG2*—had a degree ≥40, indicating central positions within the network (Figure 8B). *CD4* expression is dynamically regulated during TEX, particularly in the HCC tumor microenvironment, where TEX cells compromise immune surveillance and therapeutic efficacy [39]. *CD244*, also known as *2B4*, is a co-inhibitory receptor strongly associated with TEX. Its expression is upregulated in CD8^+^ T cells under immunosuppressive conditions, such as cytomegalovirus infection following liver transplantation [40]. *SLAMF7* plays a key role in modulating T cell function and immune escape within the HCC microenvironment. *SLAMF7* is not only involved in regulating CD8^+^ TEX but is also positively correlated with immunotherapy responsiveness in HCC patients. Its expression influences macrophage recruitment and polarization through inhibition of CCL2, thereby shaping the immune milieu [41]. *PDCD1LG2* is a critical immune checkpoint involved in TEX in HCC. Elevated *PDCD1LG2* expression is associated with favorable clinical outcomes, including improved recurrence-free, progression-free, disease-specific, and overall survival, underscoring its potential prognostic and immunomodulatory roles [42].

To identify additional molecules interacting with these TEX-related genes, we extracted interacting partners from the PPI network. Among these, we found an overlap with established markers of exhausted T cells, including *IFNG*, *CD38*, *CTLA4*, *HAVCR2*, *LAG3*, *PDCD1*, *ICOS*, *TIGIT*, and *TNFRSF18*. *CD38* had been recognized as a key surface marker of exhausted CD8^+^ T cells. These cells often co-express immunosuppressive receptors such as *PD-1* and *CTLA-4*, and exhibit impaired effector functions within the TME [43,44]. *CD38* was also implicated in regulatory T cell function, particularly in the context of tumors and autoimmune diseases [45]. *PDCD1 (PD-1)*, a central marker of TEX, is upregulated in chronic viral infections and cancers. Its expression is indicative of T cell dysfunction, and therapeutic blockade of *PD-1* could partially restore T cell activity [46,47]. Co-expression with other inhibitory receptors such as *TIGIT* and *LAG3* further enhances the exhausted phenotype [48,49]. *HAVCR2 (TIM-3)* and *LAG3* contribute to T cell inhibitory signaling and act synergistically with *PD-1*, exacerbating exhaustion [50]. *ICOS* and *TNFRSF18 (GITR)* regulate T cell activation and function. *ICOS* is associated with T cell help, while *GITR* plays a pivotal role in the activation of regulatory T cells [51,52]. *TIGIT*, as an emerging checkpoint, inhibits T cell proliferation and cytokine production via interaction with its ligand *CD155* [53,54].

Collectively, these findings indicate that the exhaustion-associated gene signature was not only centrally positioned within the PPI network but was also functionally connected to well-established markers of TEX. Their prominent roles in immune regulation and tumor progression underlines their potential as therapeutic targets in cancer immunotherapy.

## 4. Discussion

In this study, we established and validated a 26-gene TEX signature in HCC through the integration of single-cell and bulk RNA-seq data. The signature reliably stratified patients into prognostic subgroups, with the high-TEX group displaying significantly worse overall survival. Importantly, beyond prognostic stratification, our analysis demonstrated that the TEX signature was strongly correlated with immune regulatory programs, including checkpoint pathways, antigen presentation, and costimulatory signaling, emphasizing its dual value as both a prognostic biomarker and a potential guide for immunotherapeutic strategies.

Our findings align with previous studies that reported the accumulation of exhausted CD8^+^ T cells in HCC and their association with poor clinical outcomes [15]. Similar exhaustion-associated signatures have been described in recent years. For instance, Liu et al. developed a CD8 TEX signature that predicted survival in HCC [55], and Shi et al. identified an TEX signature [56]. However, our study extends these works by integrating both single-cell and bulk transcriptomic datasets, thereby capturing exhaustion at both the cellular and systemic levels.

A striking observation was that high-TEX tumors exhibited not only increased expression of inhibitory receptors (*PD-1*, *CTLA-4*, *LAG-3* and *TIM-3*) but also enhanced antigen presentation and HLA class I/II expression. This “dual immune activity” suggests that high-TEX tumors are not immunologically silent but rather represent an active yet dysfunctional immune state. Recent studies also support this concept: single-cell atlases have shown gradients of pre-exhausted and terminally exhausted T cells with preserved proliferative potential [57,58]. In addition, reviews emphasize that TEX cells remain heterogeneous and partially reinvigoratable under PD-1 blockade [59]. These findings reinforce our interpretation that high-TEX tumors, despite exhaustion, may retain immunogenic features and responsiveness to immune checkpoint inhibitors.

Checkpoint elevation in high-TEX tumors may render them susceptible to PD-1/PD-L1 blockade, while preserved antigen presentation pathways suggest a capacity for T cell reinvigoration. Such dual phenotype is reminiscent of the interferon-γ (IFN-γ)–related gene expression profile, which predicts benefit from anti–PD-1 therapy across tumors [37,60]. In HCC, exploratory biomarker analysis of atezolizumab-bevacizumab therapy similarly showed associations between response and activation of IFN-γ signaling and antigen presentation [61]. These data suggest that patients with high-TEX tumors may represent a responsive subgroup to immune checkpoint inhibitors.

Despite these insights, key questions remain unanswered. It is still unclear which TEX subsets are predictive of durable immunotherapy responses versus those that signify irreversible dysfunction. The dynamic progression from functional effectors to partially and terminally exhausted states also remains poorly defined in HCC [62].

Several limitations of our study should be acknowledged. First, the analysis was based on retrospective public datasets, which may introduce sampling bias and lack comprehensive clinical annotation. Second, although our signature was validated in multiple independent cohorts, overfitting cannot be fully excluded, underscoring the need for prospective validation. Third, our study relied primarily on computational approaches, and functional experiments are needed to establish causal links between identified genes and TEX biology. Finally, while our findings suggest that high-TEX patients may respond to immune checkpoint inhibitors, the lack of direct clinical response data prevents definitive conclusions. Addressing these limitations will be essential to translate the TEX signature into clinical decision-making.

## Supplementary Materials

The following supporting information can be downloaded at: https://www.mdpi.com/article/10.3390/genes16091034/s1. Figure S1: The significant result of 71 genes for univariate Cox regression analysis; Table S1: The result of differential analysis.
