# Supplementary material for: Bulk and Single-Cell Transcriptomes Reveal Exhausted Signature in Prognosis of Hepatocellular Carcinoma
Source: Genes (Basel). 2025 Aug 30;16(9):1034. doi: 10.3390/genes16091034 (PMC12469618; doi:10.3390/genes16091034)
Supplement: Supplementary file 1 [file genes-16-01034-s001.zip › genes-3831307-Supplementary Figure.pdf]

	pvalue	Hazard ratio
ACTR3	0.026	1.381(1.040–1.834)
SEMA4D	0.027	1.430(1.041–1.965)
CD226	0.017	2.894(1.210–6.921)
LILRB1	0.043	1.380(1.010–1.886)
LILRA1	0.008	3.183(1.346–7.528)
MGAT4A	0.030	1.342(1.029–1.750)
GBP5	0.005	1.275(1.075–1.512)
SPATA2L	0.027	0.720(0.539–0.963)
MRPL55	0.034	0.779(0.619–0.981)
PYHIN1	0.003	1.679(1.193–2.362)
CDPF1	0.047	0.734(0.541–0.996)
CLEC12A	0.003	2.064(1.280–3.329)
ECI1	0.004	0.664(0.503–0.877)
PAPD4	0.045	1.480(1.009–2.171)
GIMAP7	<0.001	1.380(1.141–1.669)
SAMD9L	0.034	1.282(1.019–1.614)
TPCN2	0.049	1.242(1.001–1.540)
FGD2	0.014	1.713(1.117–2.626)
ARHGAP30	0.028	1.273(1.027–1.577)
BLOC1S6	0.047	1.466(1.006–2.138)
GBP1	0.047	1.184(1.002–1.398)
LAMTOR2	0.041	0.765(0.592–0.989)
HLA−DQA1	0.035	1.158(1.010–1.327)
JAK2	0.020	1.389(1.054–1.831)
NRROS	0.029	1.392(1.034–1.873)
LCP2	0.007	1.370(1.090–1.723)
MYH10	0.010	1.311(1.066–1.613)
GIMAP6	0.039	1.279(1.013–1.614)
NT5E	0.050	1.171(1.000–1.370)
OXCT1	0.005	1.287(1.080–1.533)
BOLA1	0.043	0.750(0.568–0.992)
CD244	0.010	1.707(1.137–2.562)
RAB8B	0.042	1.284(1.009–1.634)
TPCN1	0.034	0.758(0.586–0.980)
FBXW5	0.035	0.726(0.539–0.978)
TOLLIP	0.014	0.612(0.414–0.906)
CROT	0.019	1.267(1.040–1.543)
GIMAP4	0.009	1.307(1.070–1.597)
MIS18BP1	0.046	1.368(1.005–1.862)
PRF1	0.024	1.233(1.028–1.478)
SRGN	0.009	1.235(1.055–1.444)
AGK	0.023	1.535(1.061–2.220)
TMEM30A	0.037	1.391(1.020–1.898)
PRKCH	0.007	1.558(1.132–2.145)
SLAMF7	0.014	1.235(1.044–1.459)
PTPRC	0.008	1.266(1.065–1.505)
SELL	0.022	1.242(1.031–1.495)
SAMSN1	0.008	1.393(1.091–1.778)
SPN	0.038	1.368(1.017–1.840)
STAT1	0.042	1.173(1.006–1.368)
VCAM1	0.001	1.220(1.082–1.374)
WIPF1	0.009	1.310(1.069–1.606)
PPDPF	0.015	0.851(0.747–0.969)
ZNF672	0.046	0.714(0.513–0.995)
PDCD1LG2	0.028	1.330(1.031–1.715)
AKNA	0.005	1.423(1.110–1.823)
EOMES	0.003	1.672(1.193–2.343)
TMEM222	0.021	0.659(0.463–0.939)
LRRC8C	0.020	1.630(1.079–2.463)
L3MBTL3	0.031	1.471(1.036–2.088)
FAM126A	0.026	1.605(1.059–2.434)
ZC3H10	0.019	0.396(0.183–0.858)
CD164	0.025	1.386(1.042–1.843)
SUCLA2	0.034	1.366(1.024–1.822)
CD3G	0.009	1.462(1.099–1.944)
CD4	0.006	1.267(1.071–1.500)
RCSD1	0.027	1.351(1.034–1.765)
HNRNPLL	0.043	1.528(1.014–2.304)
FBXO44	0.038	0.765(0.594–0.986)
LPXN	0.012	1.403(1.078–1.828)
ZEB2	0.034	1.477(1.030–2.118)

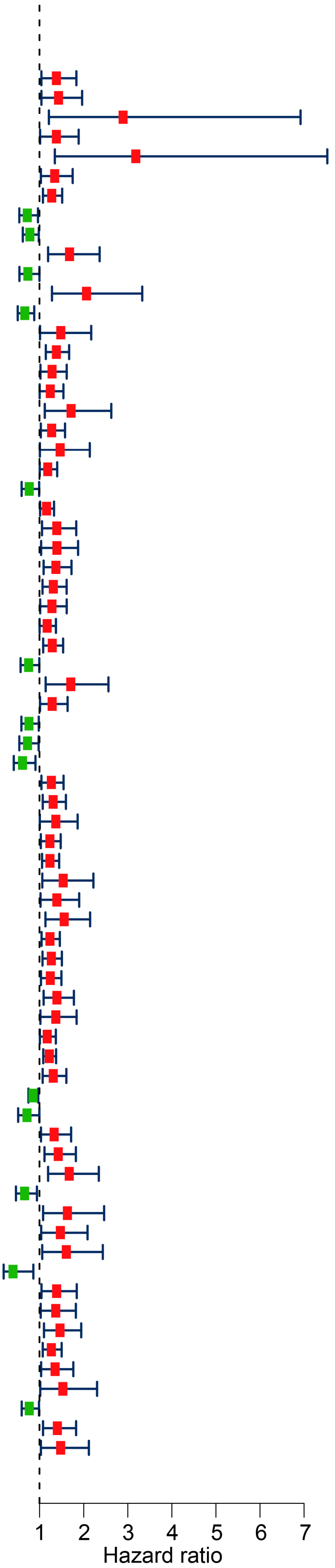

Supplementary Figure S1. The significant result of 71 genes for univariate Cox regression analysis. The red and green box represent positive and negative association with the risk event, respectively.
